# Interpositional Arthroplasty with Iliotibial Band Autograft Fixation to the Proximal Phalanx: Clinical Outcomes in Advanced Hallux Rigidus

**DOI:** 10.3390/medicina62081537

**Published:** 2026-08-10

**Authors:** Furkan Soy, Yasin Kozan, Ozan Pehlivan, Sancar Serbest

**Affiliations:** 1 Department of Orthopaedics and Traumatology, Faculty of Medicine, Kırıkkale University, Kırıkkale 71450, Türkiye; pehlivanozan@gmail.com (O.P.); dr.sancarserbest@gmail.com (S.S.); 2 Department of Orthopaedics and Traumatology, Akdağmadeni State Hospital, Yozgat 66300, Türkiye; yasinkozan.dr@gmail.com

**Keywords:** hallux rigidus, ınterpositional arthroplasty, ıliotibial band autograft, great toe arthritis, biological resurfacing

## Abstract

*Background and Objectives:* Hallux rigidus is a degenerative condition of the first metatarsophalangeal joint that causes pain and limitation of motion. The aim of this study was to evaluate the clinical outcomes of a modified interpositional arthroplasty technique using an iliotibial band autograft fixed to the proximal phalanx in patients with advanced hallux rigidus. *Materials and Methods:* This prospective observational case series included 19 patients with Coughlin–Shurnas Grade III or IV hallux rigidus who underwent surgery between February 2023 and February 2025. Clinical outcomes were assessed using Visual Analog Scale (VAS), American Orthopaedic Foot and Ankle Society (AOFAS), and range-of-motion (ROM) measurements preoperatively and at postoperative 1, 6, and 12 months. *Results:* The mean VAS score decreased from 9.0 ± 0.82 preoperatively to 1.58 ± 0.51 at 12 months (*p* < 0.001). The mean AOFAS score increased from 35.1 ± 2.7 to 74.8 ± 10.9 (*p* = 0.001). ROM improved significantly at 1 and 6 months; however, the 12-month ROM was not significantly different from the preoperative value (36.3° ± 5.6° vs. 31.0° ± 5.5°, *p* = 0.082). *Conclusions:* Interpositional arthroplasty using an iliotibial band autograft fixed to the proximal phalanx was associated with short-term reductions in pain and improvements in functional scores. However, the early increase in range of motion was not maintained to the same extent at 12 months.

## 1. Introduction

Hallux rigidus is a progressive degenerative disorder of the first metatarsophalangeal (MTP) joint characterized by pain and restricted motion. It is the second most common disorder of the first MTP joint after hallux valgus [1]. Its etiology is multifactorial and often remains idiopathic. Proposed contributing factors include acute or repetitive trauma, osteochondral injury, anatomical or biomechanical abnormalities of the first ray, familial predisposition, and secondary degeneration associated with inflammatory or metabolic arthropathies [1]. Typical findings include restricted dorsiflexion, dorsal osteophyte formation, and functional impairment in daily activities [1]. In advanced disease, nonoperative treatment is frequently insufficient, and surgery may be indicated [2].

Various surgical techniques have been described for the treatment of hallux rigidus, including cheilectomy, osteotomies, resection arthroplasty, interpositional arthroplasty, implant arthroplasty, and arthrodesis [2,3]. Among these procedures, arthrodesis has traditionally been considered the reference standard because of its high success rates and reliable pain relief [1,4]. However, it eliminates joint motion, may restrict footwear choices, and alters forefoot biomechanics [4].

For this reason, there has been increasing interest in joint-preserving surgical options, particularly in active patients and individuals who wish to maintain joint mobility. Interpositional arthroplasty has emerged as a method aimed at reducing pain and preserving joint motion by placing a biological or synthetic material between the joint surfaces [2]. Recent systematic reviews have reported that interpositional arthroplasty is associated with significant improvements in pain and functional outcomes; however, long-term findings remain heterogeneous, and the overall level of evidence is limited [5,6].

Materials used in interpositional arthroplasty include capsular tissues, tendon grafts, allografts, and synthetic materials, with autologous soft tissue offering advantages in terms of biocompatibility and lower complication rates [6]. However, in most techniques described in the literature, the graft is typically placed over the first metatarsal head and fixed using various methods [7].

In the present study, a modified surgical technique was applied in which, unlike previously described methods, an iliotibial band autograft was used for interpositional arthroplasty, and the graft was placed on the articular surface of the proximal phalanx rather than the first metatarsal and fixed using bone tunnels.

The aim of this study was to evaluate the clinical outcomes of first MTP interpositional arthroplasty performed using an iliotibial band autograft and to investigate the effects of this modified technique on pain, function, and range of motion.

## 2. Materials and Methods

### 2.1. Study Design

In this prospective observational case series, patients who underwent first metatarsophalangeal (MTP) joint interpositional arthroplasty using an iliotibial band (ITB) autograft for advanced hallux rigidus between February 2023 and February 2025 were included. All procedures were performed at a single center using a standardized surgical technique by two orthopedic surgeons with expertise in foot and ankle surgery. The study protocol was approved by the Kırıkkale University Clinical Research Ethics Committee (approval code: 01/01; approval date: 12 January 2023), and the study was conducted in accordance with the principles of the Declaration of Helsinki.

All patients completed the predefined postoperative 12-month clinical assessment. The 12-month assessment was designated as the standardized final clinical endpoint, whereas the longitudinal analyses of VAS, AOFAS, and ROM included the preoperative, 1-month, 6-month, and 12-month evaluations. The mean overall clinical follow-up duration was 14.7 ± 3.8 months, with a range of 12–21 months. Complications, reoperations, and conversions to arthrodesis were recorded throughout each patient’s entire available follow-up period.

### 2.2. Patient Selection

**Figure 1:**
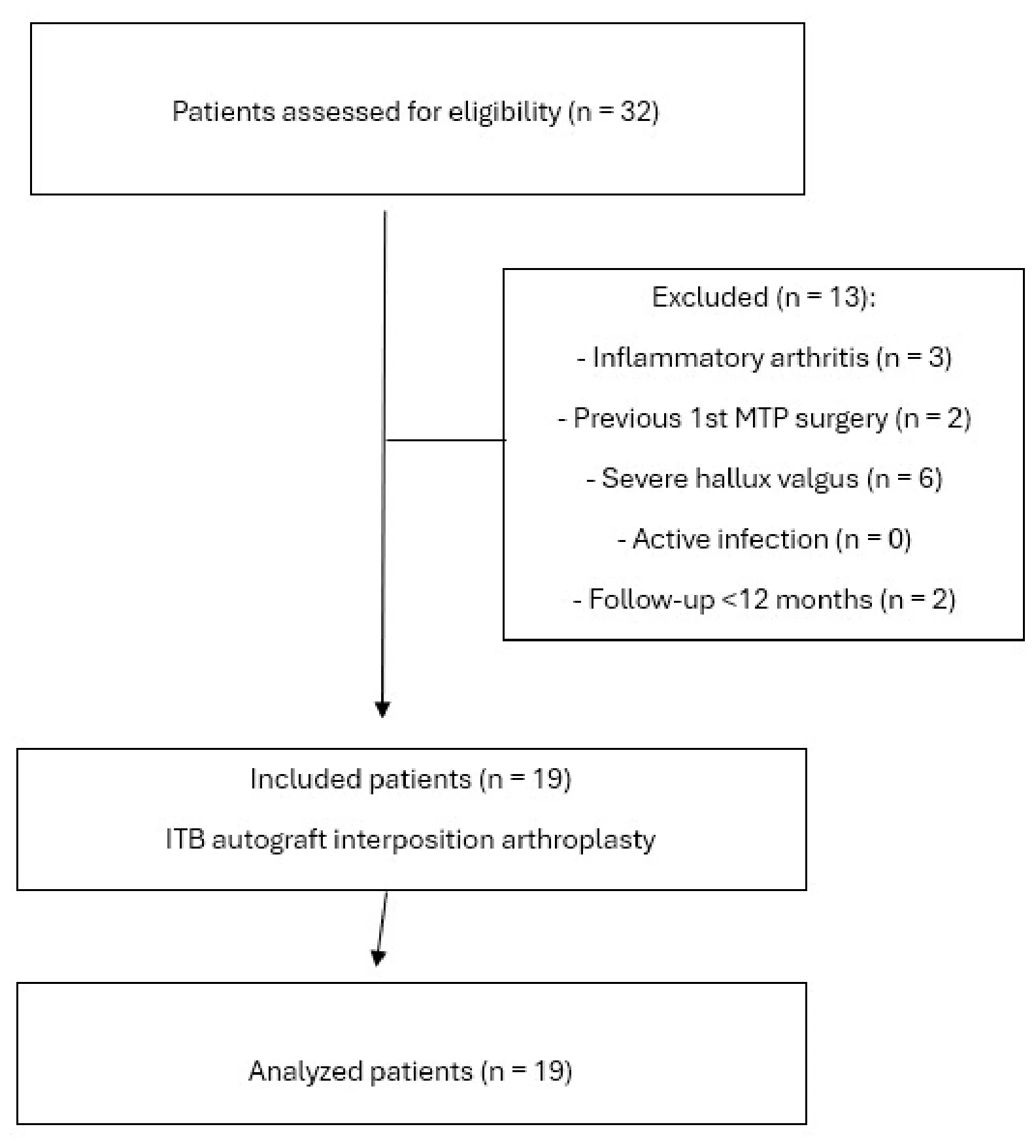
Flow diagram of the patient selection process.

The inclusion criteria were the presence of symptomatic hallux rigidus unresponsive to conservative treatment, advanced-stage disease, and a minimum follow-up of 12 months. The exclusion criteria were the presence of inflammatory arthritis, active infection, severe hallux valgus deformity, a history of previous surgery involving the first MTP joint, and incomplete clinical data or insufficient follow-up duration. A flow diagram of the patient selection process is presented in Figure 1.

All patients were evaluated clinically and radiographically before surgery. Disease severity was classified according to the Coughlin–Shurnas classification. The study cohort included patients with Grade III and Grade IV hallux rigidus. The indication for surgery was determined based on persistent pain despite conservative treatment, functional limitation, and decreased range of motion.

General medical comorbidities were not included as predefined study variables and were therefore not analyzed quantitatively.

### 2.3. Surgical Procedure

**Figure 2:**
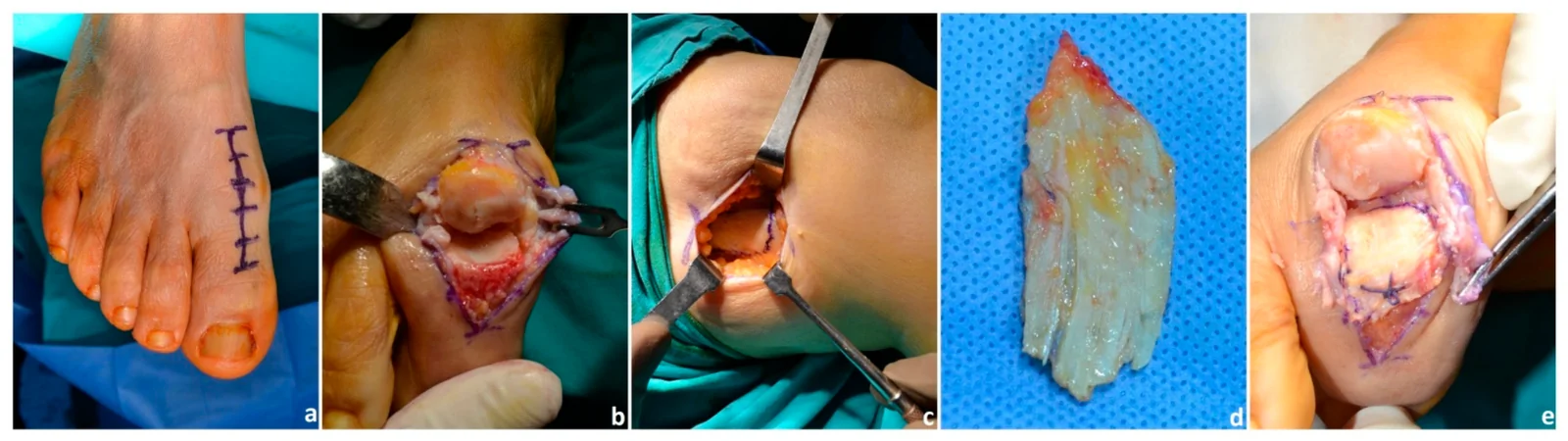
Surgical steps for joint exposure, iliotibial band autograft harvesting, and graft preparation and placement. (**a**) Dorsal approach to the first metatarsophalangeal joint. (**b**) Excision of dorsal osteophytes and joint preparation. (**c**) Harvesting of the iliotibial band autograft from the lateral aspect of the knee. (**d**) Preparation of the graft without folding, preserving its native thickness. (**e**) Placement of the graft onto the articular surface of the proximal phalanx.

All surgical procedures were performed in the supine position under regional or general anesthesia with the use of a pneumatic tourniquet. Routine antibiotic prophylaxis was administered preoperatively in all cases. A standard dorsal approach to the first MTP joint was used (Figure 2a). After opening the joint capsule, dorsal osteophytes were excised and minimal debridement was performed while preserving the existing bone stock (Figure 2b).

As the interposition material, an approximately 4–5 cm-long iliotibial band autograft was harvested through a separate incision from the lateral side of the ipsilateral knee (Figure 2c). The graft was prepared without folding, preserving its natural thickness (Figure 2d). Unlike techniques described in the literature, the graft was placed directly onto the articular surface of the proximal phalanx rather than the first metatarsal head (Figure 2e).

**Figure 3:**
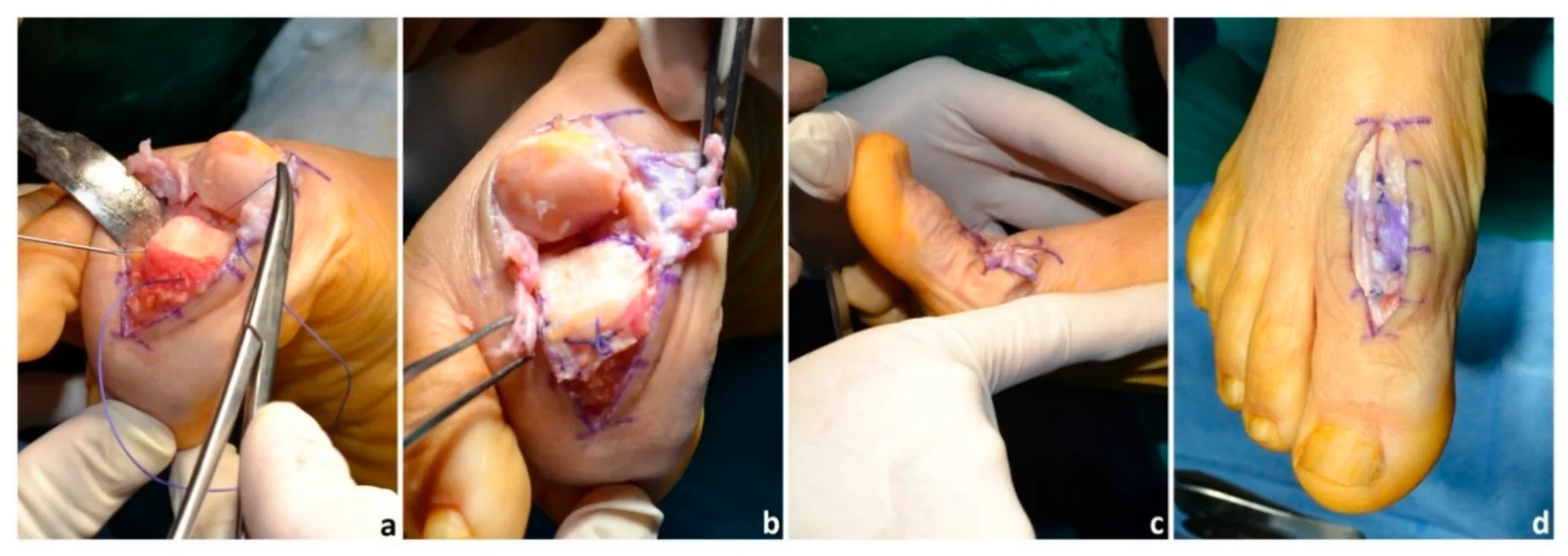
Graft fixation and final intraoperative appearance. (**a**) Creation of bone tunnels in the proximal phalanx. (**b**) Fixation of the graft with absorbable sutures. (**c**) Assessment of joint congruity and passive range of motion. (**d**) Closure of the joint capsule.

Bone tunnels were created in the proximal phalanx, and the graft was fixed using absorbable sutures (Figure 3a). The graft was tensioned and positioned to provide homogeneous coverage of the proximal phalangeal articular surface (Figure 3b). Intraoperatively, joint congruity and passive range of motion were assessed (Figure 3c). The capsule and soft tissues were anatomically closed (Figure 3d). The iliotibial band was selected to provide an autologous graft of sufficient size and thickness for single-layer coverage of the proximal phalangeal articular surface and fixation through bone tunnels.

Postoperative analgesia consisted of paracetamol three times daily for 5 days, with dexketoprofen twice daily as needed for up to 5 days. No routine postoperative antibiotic therapy was administered beyond preoperative antibiotic prophylaxis. For venous thromboembolism prophylaxis, enoxaparin 0.4 mL was administered subcutaneously once daily for 2 weeks. No postoperative shoe or orthosis was used. Patients remained non-weight-bearing for the first 2 postoperative weeks, while active and passive range-of-motion exercises of the first MTP joint were initiated immediately after surgery. Sutures were removed at the 2-week follow-up. Thereafter, full weight-bearing and transition to regular footwear were permitted as tolerated. Formal physiotherapy was not routinely prescribed. Patients were generally permitted to resume unrestricted daily activities and sports at approximately 6 weeks, depending on their clinical recovery.

### 2.4. Clinical Evaluation

Clinical outcomes were evaluated using data routinely recorded during preoperative assessment and postoperative follow-up visits at 1, 6, and 12 months. Pain was assessed using the Visual Analog Scale (VAS), and functional outcomes were evaluated using the American Orthopaedic Foot and Ankle Society (AOFAS) hallux metatarsophalangeal-interphalangeal score. Range of motion (ROM) of the first MTP joint, including dorsiflexion and plantar flexion angles, was measured with a goniometer and recorded in degrees (°). All measurements were obtained by the same observer using a standardized clinical protocol under comparable conditions.

Postoperative complications and reoperations were evaluated through review of medical records and follow-up examinations. The assessed events included surgical-site wound problems, infection, persistent pain or joint stiffness, transfer metatarsalgia, symptomatic instability, donor-site morbidity, reoperation, and conversion to first metatarsophalangeal joint arthrodesis. Donor-site morbidity was assessed based on clinically documented pain, wound-related problems, or other complaints involving the lateral knee region from which the iliotibial band autograft was harvested.

### 2.5. Statistical Analysis

Statistical analyses were performed using SPSS version 20.0 (IBM Corp., Armonk, NY, USA). Continuous variables were summarized as mean ± standard deviation. The distributions of the repeated VAS, AOFAS, and ROM measurements were evaluated using the Shapiro–Wilk test. Because the assumptions required for parametric repeated-measures analysis were not consistently satisfied and the sample size was limited, changes across the preoperative, 1-month, 6-month, and 12-month assessments were analyzed using the Friedman test. When a significant overall time effect was identified, pairwise comparisons were performed using the Wilcoxon signed-rank test with Bonferroni’s adjustment for six comparisons. The mean values at each assessment time point were reported with corresponding 95% confidence intervals. All tests were two-sided, and an adjusted *p*-value of <0.05 was considered statistically significant.

## 3. Results

A total of 19 patients were included in the study, and the mean age of the patients was 60.3 ± 4.0 years. Twelve patients (63.2%) were female and seven (36.8%) were male. The operated side was right in 11 patients (57.9%) and left in 8 patients (42.1%). According to the Coughlin–Shurnas classification, 12 patients (63.2%) had Grade III hallux rigidus and 7 patients (36.8%) had Grade IV hallux rigidus (Table 1). All patients completed the planned postoperative 12-month clinical assessment. The mean overall clinical follow-up duration was 14.7 ± 3.8 months, the median follow-up duration was 16 months, and the range was 12–21 months. The 12-month assessment represented the standardized final clinical endpoint, whereas the longitudinal analyses included the preoperative, 1-month, 6-month, and 12-month VAS, AOFAS, and ROM measurements. Complications and reoperations were assessed until each patient’s most recent available follow-up.

In pain assessment, the mean preoperative VAS score was 9.0 ± 0.82, whereas it was 3.89 ± 0.81, 1.53 ± 0.51, and 1.58 ± 0.51 at postoperative 1, 6, and 12 months, respectively (Table 2). VAS scores were significantly lower at all postoperative assessments than preoperatively (adjusted *p* < 0.001 for all comparisons).

In functional assessment, the mean preoperative AOFAS score was 35.1 ± 2.7, whereas it was 75.0 ± 10.2, 80.1 ± 3.3, and 74.8 ± 10.9 at postoperative 1, 6, and 12 months, respectively (Table 3). AOFAS scores were significantly higher at all postoperative assessments than at the preoperative assessment (adjusted *p* < 0.001 at 1 and 6 months and adjusted *p* = 0.001 at 12 months).

The mean preoperative ROM was 31.0° ± 5.5° and increased to 58.3° ± 5.6° at 1 month and 55.3° ± 6.0° at 6 months (adjusted *p* < 0.001 for both comparisons) (Table 4). At 12 months, the mean ROM was 36.3° ± 5.6°, which was not significantly different from the preoperative value (adjusted *p* = 0.082).

During the postoperative follow-up period, wound-related complications occurred in three patients (15.8%), and infection was documented in one patient (5.3%). Persistent pain or joint stiffness was recorded in one patient (5.3%). No patient developed transfer metatarsalgia or symptomatic instability. Donor-site morbidity related to iliotibial band harvesting was documented in one patient (5.3%). Two patients (10.5%) underwent reoperation, and one patient (5.3%) required conversion to first metatarsophalangeal joint arthrodesis. Apart from the one patient who underwent conversion to arthrodesis, no additional definitive clinical failures were documented.

## 4. Discussion

The principal findings of this study were short-term reductions in pain and improvements in functional scores; however, the early gain in range of motion was not maintained to the same extent at 12 months. These results are consistent with recent reviews and systematic analyses reporting a renewed interest in interpositional arthroplasty as a joint-preserving option in the treatment of hallux rigidus [1,2,8,9]. Although arthrodesis remains the reference standard for advanced hallux rigidus, it may not be suitable for all patients because it eliminates joint motion, may limit footwear choices, and alters forefoot biomechanics [1,9,10,11,12]. Given the small sample size and the absence of a comparator group, these findings should be interpreted as preliminary evidence of short-term clinical improvement rather than evidence of equivalence or superiority to arthrodesis or other surgical procedures.

The rationale for interpositional arthroplasty is to place a biological or biocompatible layer between degenerative joint surfaces to reduce pain while preserving some degree of joint motion. The classical capsular interposition technique described by Hamilton et al. formed the basis of this approach, and subsequent studies have introduced various soft-tissue-based modifications [13,14,15,16,17]. The feasibility of joint-preserving surgery has been demonstrated using autografts, allografts, and different capsular-tendinous materials [13,14,15,16,17,18,19,20,21,22,23,24,25]. This wide variety of techniques described in the literature indicates that interpositional arthroplasty is not a single standardized procedure and that outcomes largely depend on the graft material used, the extent of resection, soft tissue balance, and the fixation technique [2,8,19].

In our series, the marked decrease in VAS scores and the significant increase in AOFAS scores are consistent with the trends reported in the literature regarding pain relief and functional improvement after interpositional arthroplasty. Emmons and Carreira, in their systematic review including 20 studies and 539 feet, reported clinically significant improvement in most studies using standardized scoring systems, with a low rate of progression to further surgical intervention [2]. Similarly, the meta-analysis by Patel et al. demonstrated significant improvements in mean AOFAS scores along with increases in dorsiflexion and overall range of motion [8].

However, the absence of a statistically significant difference between the preoperative and 12-month range-of-motion values, despite significant improvements at 1 month and 6 months, is clinically important. The early postoperative gain in range of motion was not maintained to the same extent at 12 months. Because maintenance of motion is a principal rationale for selecting interpositional arthroplasty, this finding represents an important limitation of the observed short-term outcome and should be clearly communicated during patient counseling. Previous capsular interposition series have similarly reported early improvements in motion, whereas studies with longer follow-up have shown a gradual reduction in motion or functional gains that exceed the absolute improvement in range of motion [14,16,22]. Other long-term follow-up studies suggest that patient satisfaction and pain relief may be maintained even when normal range of motion is not restored [25]. Nevertheless, the present findings are limited to standardized 12-month outcome measurements and should not be extrapolated to the long-term performance or durability of the technique.

A unique aspect of our study is that the interpositional arthroplasty technique was modified not only in terms of the graft material used but also in the placement and fixation of the graft. In most techniques described in the literature, the graft is either used as a dorsal capsular flap based on the extensor hallucis brevis or positioned to resurface the metatarsal side [4,13,14,15,17,18,20,23,24]. A previous meta-analysis reported higher postoperative AOFAS scores in studies using autografts than in those using allografts and suggested that preservation of the short flexors may benefit plantarflexion and push-off strength [8]. In our technique, an approximately 4–5 cm iliotibial band autograft with preserved natural thickness was harvested from the lateral knee and placed not on the metatarsal head but directly onto the articular surface of the proximal phalanx, where it was fixed using bone tunnels and absorbable sutures. The graft was tensioned and secured over the proximal phalangeal surface to obtain consistent coverage of the articular surface. Bone-tunnel fixation was intended to maintain graft position; however, this represents a technical rationale for the procedure rather than evidence of superior graft stability or clinical performance. This approach differs from conventional metatarsal-based techniques and constitutes the surgical novelty of our study. Accordingly, the present findings support the technical feasibility of proximal phalangeal graft placement and fixation. However, because no alternative interpositional technique was included as a comparator, the current study cannot determine whether proximal phalangeal placement or fixation provides an advantage over other graft materials, graft locations, or fixation methods. Graft position, displacement, biological integration, and structural durability were not objectively evaluated using biomechanical testing, serial imaging, or second-look assessment. Therefore, the present study cannot establish whether bone-tunnel fixation provides a clinical or biomechanical advantage over alternative fixation methods or interposition techniques without formal fixation.

The iliotibial band was selected to provide an autologous graft with sufficient dimensions and relatively consistent thickness to cover the proximal phalangeal articular surface as a single layer and to permit fixation through bone tunnels without relying solely on the available local capsular tissue. This represents the technical rationale for the graft choice rather than evidence of superiority. The novelty of the present technique does not lie solely in the use of fascia-based tissue, which has previously been described for first MTP interpositional arthroplasty, but in the combination of an iliotibial band autograft, placement over the proximal phalangeal articular surface, and fixation through bone tunnels. However, because the present study did not compare the iliotibial band with local capsular interposition, tendon autografts, allografts, or synthetic materials, no conclusion can be drawn regarding the relative biological, mechanical, or clinical advantage of the selected graft.

However, harvesting the iliotibial band requires a second operative site and introduces additional surgical burden, including a separate incision, scarring, donor-site pain, and potential wound-related problems. In the present series, donor-site morbidity was documented in one patient (4.3%); however, donor-site outcomes were assessed retrospectively, and minor or transient symptoms may have been underreported. Because the study did not include a comparator group using local capsular tissue or another graft source, it cannot determine whether the potential technical benefits of adequate graft dimensions and bone-tunnel fixation outweigh the additional morbidity associated with a separate donor-site procedure. This trade-off should be considered during patient selection and preoperative counseling.

Randomized studies and long-term follow-up data have shown that arthrodesis provides reliable pain relief and high patient satisfaction in advanced hallux rigidus [10,11]. Because the present study did not include an arthrodesis group, the findings should not be interpreted as evidence that the proposed technique is equivalent or superior to arthrodesis. Rather, this study describes the short-term clinical outcomes of a modified interpositional arthroplasty technique in a selected patient cohort. Arthrodesis remains an established treatment for advanced hallux rigidus, and prospective comparative studies are required to determine the relative indications, benefits, limitations, and durability of the present procedure. Importantly, use of this technique does not preclude subsequent salvage arthrodesis in the event of clinical failure.

This study has several limitations. First, the absence of a control group and the relatively small sample size limit the generalizability of the findings. General medical comorbidities were not systematically collected as predefined study variables.

The VAS, AOFAS, and range-of-motion outcomes in this study were based on standardized 12-month assessments. Although the overall clinical follow-up ranged from 12 to 21 months, the available duration remains insufficient to evaluate the long-term durability of symptomatic improvement, graft performance, progression of degenerative changes, or the late risk of revision surgery and conversion to arthrodesis. The study also has several strengths. A standardized surgical technique was used in all patients at a single center by surgeons with expertise in foot and ankle surgery. Moreover, the study describes a technically distinct modification involving fixation of an iliotibial band autograft to the proximal phalangeal articular surface, an approach that has been infrequently reported in the literature.

## 5. Conclusions

Interpositional arthroplasty using an iliotibial band autograft fixed to the proximal phalanx was associated with short-term reductions in pain and improvements in functional scores. However, the early increase in range of motion was not maintained to the same extent at 12 months.

## Figures and Tables

**Table 1:** Demographic and Clinical Characteristics of the Included Patients.

Variable	Value
Age (mean ± SD)	60.3 ± 4.0
Age range (min–max)	51–67
Sex	Female: 12 (%63.2)/Male: 7 (%36.8)
Operated side	Right: 11 (%57.9)/Left: 8 (%42.1)
Coughlin–Shurnas Grade	Grade III: 12 (63.2%)/Grade IV: 7 (36.8%)

**Table 2:** Time-Dependent Changes in VAS Scores after Interpositional Arthroplasty using an Iliotibial Band Autograft.

Time	VAS (Mean ± SD)	95% CI	*p* *
Preoperative	9.0 ± 0.82	8.60–9.40	-
1 Month	3.89 ± 0.81	3.50–4.28	<0.001
6 Months	1.53 ± 0.51	1.28–1.78	<0.001
12 Months	1.58 ± 0.51	1.33–1.83	<0.001

* Overall changes over time were evaluated using the Friedman test. Post hoc pairwise comparisons were performed using the Wilcoxon signed-rank test with Bonferroni’s adjustment for six comparisons. The displayed *p*-values represent adjusted comparisons between each postoperative assessment and the preoperative value. The 95% confidence intervals refer to the mean at each assessment time point.

**Table 3:** Time-Dependent Changes in AOFAS Scores after Interpositional Arthroplasty using an Iliotibial Band Autograft.

Time	AOFAS (Mean ± SD)	95% CI	*p* *
Preoperative	35.1 ± 2.7	33.93–36.27	-
1 Month	75.0 ± 10.2	70.59–79.41	<0.001
6 Months	80.1 ± 3.3	78.67–81.53	<0.001
12 Months	74.8 ± 10.9	70.09–79.51	0.001

* Overall changes over time were evaluated using the Friedman test. Post hoc pairwise comparisons were performed using the Wilcoxon signed-rank test with Bonferroni’s adjustment for six comparisons. The displayed *p*-values represent adjusted comparisons between each postoperative assessment and the preoperative value. The 95% confidence intervals refer to the mean at each assessment time point.

**Table 4:** Time-Dependent Changes in Range of Motion of the MTP Joint.

Time	ROM (°) (Mean ± SD)	95% CI	*p* *
Preoperative	31.0 ± 5.5	28.35–33.65	-
1 Month	58.3 ± 5.6	55.60–61.00	<0.001
6 Months	55.3 ± 6.0	52.41–58.19	<0.001
12 Months	36.3 ± 5.6	33.60–39.00	0.082

* Overall changes over time were evaluated using the Friedman test. Post hoc pairwise comparisons were performed using the Wilcoxon signed-rank test with Bonferroni’s adjustment for six comparisons. The displayed *p*-values represent adjusted comparisons between each postoperative assessment and the preoperative value. The 95% confidence intervals refer to the mean at each assessment time point.

## Data Availability

The data supporting the findings of this study are available from the corresponding author upon reasonable request.

## Abbreviations

The following abbreviations are used in this manuscript:

AOFAS	American Orthopaedic Foot and Ankle Society
CI	Confidence interval
ITB	Iliotibial band
MTP	Metatarsophalangeal
ROM	Range of motion
SD	Standard deviation
SPSS	Statistical Package for the Social Sciences
VAS	Visual Analog Scale
